# Supplementary material for: Resolving the full spectrum of human genome variation using Linked-Reads
Source: Genome Res. 2019 Apr;29(4):635–45. doi: 10.1101/gr.234443.118 (PMC6442396; doi:10.1101/gr.234443.118)
Supplement: Supplemental Material [file supp_gr.234443.118_Supplemental_longranger-2.2.zip › longranger-2.2.tar.bz2!/lr2.2-public/lib/stan/cmdstan/stan/doc/api/html/classstan_1_1mcmc_1_1base__nuts-members.html]

Stan: Member List


|  |  |
| --- | --- |
|  | Stan  2.5.0  probability, sampling & optimization |


- Main Page
- Related Pages
- Namespaces
- Classes
- Files

- Class List
- Class Index
- Class Hierarchy
- Class Members

All Classes Namespaces Files Functions Variables Typedefs Enumerations Enumerator Friends Macros Pages

- stan
- mcmc
- base\_nuts

stan::mcmc::base\_nuts< M, P, H, I, BaseRNG > Member List

This is the complete list of members for stan::mcmc::base\_nuts< M, P, H, I, BaseRNG >, including all inherited members.

|  |  |  |
| --- | --- | --- |
| base\_hmc(M &m, BaseRNG &rng, std::ostream \*o, std::ostream \*e) | stan::mcmc::base\_hmc< M, P, H, I, BaseRNG > | inline |
| base\_mcmc(std::ostream \*o, std::ostream \*e) | stan::mcmc::base\_mcmc | inline |
| base\_nuts(M &m, BaseRNG &rng, std::ostream \*o, std::ostream \*e) | stan::mcmc::base\_nuts< M, P, H, I, BaseRNG > | inline |
| build\_tree(int depth, Eigen::VectorXd &rho, ps\_point \*z\_init\_parent, ps\_point &z\_propose, nuts\_util &util) | stan::mcmc::base\_nuts< M, P, H, I, BaseRNG > | inline |
| compute\_criterion(ps\_point &start, P &finish, Eigen::VectorXd &rho)=0 | stan::mcmc::base\_nuts< M, P, H, I, BaseRNG > | pure virtual |
| depth\_ | stan::mcmc::base\_nuts< M, P, H, I, BaseRNG > |  |
| epsilon\_ | stan::mcmc::base\_hmc< M, P, H, I, BaseRNG > | protected |
| epsilon\_jitter\_ | stan::mcmc::base\_hmc< M, P, H, I, BaseRNG > | protected |
| err\_stream\_ | stan::mcmc::base\_mcmc | protected |
| get\_current\_stepsize() | stan::mcmc::base\_hmc< M, P, H, I, BaseRNG > | inline |
| get\_max\_delta() | stan::mcmc::base\_nuts< M, P, H, I, BaseRNG > | inline |
| get\_max\_depth() | stan::mcmc::base\_nuts< M, P, H, I, BaseRNG > | inline |
| get\_nominal\_stepsize() | stan::mcmc::base\_hmc< M, P, H, I, BaseRNG > | inline |
| get\_sampler\_diagnostic\_names(std::vector< std::string > &model\_names, std::vector< std::string > &names) | stan::mcmc::base\_hmc< M, P, H, I, BaseRNG > | inlinevirtual |
| get\_sampler\_diagnostics(std::vector< double > &values) | stan::mcmc::base\_hmc< M, P, H, I, BaseRNG > | inlinevirtual |
| get\_sampler\_param\_names(std::vector< std::string > &names) | stan::mcmc::base\_nuts< M, P, H, I, BaseRNG > | inlinevirtual |
| get\_sampler\_params(std::vector< double > &values) | stan::mcmc::base\_nuts< M, P, H, I, BaseRNG > | inlinevirtual |
| get\_stepsize\_jitter() | stan::mcmc::base\_hmc< M, P, H, I, BaseRNG > | inline |
| hamiltonian\_ | stan::mcmc::base\_hmc< M, P, H, I, BaseRNG > | protected |
| init\_stepsize() | stan::mcmc::base\_hmc< M, P, H, I, BaseRNG > | inline |
| integrator\_ | stan::mcmc::base\_hmc< M, P, H, I, BaseRNG > | protected |
| max\_delta\_ | stan::mcmc::base\_nuts< M, P, H, I, BaseRNG > |  |
| max\_depth\_ | stan::mcmc::base\_nuts< M, P, H, I, BaseRNG > |  |
| n\_divergent\_ | stan::mcmc::base\_nuts< M, P, H, I, BaseRNG > |  |
| n\_leapfrog\_ | stan::mcmc::base\_nuts< M, P, H, I, BaseRNG > |  |
| name() | stan::mcmc::base\_mcmc | inline |
| name\_ | stan::mcmc::base\_mcmc | protected |
| nom\_epsilon\_ | stan::mcmc::base\_hmc< M, P, H, I, BaseRNG > | protected |
| out\_stream\_ | stan::mcmc::base\_mcmc | protected |
| rand\_int\_ | stan::mcmc::base\_hmc< M, P, H, I, BaseRNG > | protected |
| rand\_uniform\_ | stan::mcmc::base\_hmc< M, P, H, I, BaseRNG > | protected |
| sample\_stepsize() | stan::mcmc::base\_hmc< M, P, H, I, BaseRNG > | inline |
| seed(const Eigen::VectorXd &q) | stan::mcmc::base\_hmc< M, P, H, I, BaseRNG > | inline |
| set\_max\_delta(const double d) | stan::mcmc::base\_nuts< M, P, H, I, BaseRNG > | inline |
| set\_max\_depth(const int d) | stan::mcmc::base\_nuts< M, P, H, I, BaseRNG > | inline |
| set\_nominal\_stepsize(const double e) | stan::mcmc::base\_hmc< M, P, H, I, BaseRNG > | inlinevirtual |
| set\_stepsize\_jitter(const double j) | stan::mcmc::base\_hmc< M, P, H, I, BaseRNG > | inlinevirtual |
| transition(sample &init\_sample) | stan::mcmc::base\_nuts< M, P, H, I, BaseRNG > | inlinevirtual |
| write\_sampler\_param\_names(std::ostream &o) | stan::mcmc::base\_nuts< M, P, H, I, BaseRNG > | inlinevirtual |
| write\_sampler\_params(std::ostream &o) | stan::mcmc::base\_nuts< M, P, H, I, BaseRNG > | inlinevirtual |
| write\_sampler\_state(std::ostream \*o) | stan::mcmc::base\_hmc< M, P, H, I, BaseRNG > | inlinevirtual |
| z() | stan::mcmc::base\_hmc< M, P, H, I, BaseRNG > | inline |
| z\_ | stan::mcmc::base\_hmc< M, P, H, I, BaseRNG > | protected |
| ~base\_mcmc() | stan::mcmc::base\_mcmc | inlinevirtual |
| ~base\_nuts() | stan::mcmc::base\_nuts< M, P, H, I, BaseRNG > | inline |


---

[ Stan Home Page ]

*© 2011–2014,
Stan Development Team.*
